# Apps for Mental Health: An Evaluation of Behavior Change Strategies and Recommendations for Future Development

**DOI:** 10.3389/frai.2019.00030

**Published:** 2019-12-17

**Authors:** Felwah Alqahtani, Ghazayil Al Khalifah, Oladapo Oyebode, Rita Orji

**Affiliations:** ^1^Computer Science, Dalhousie University, Halifax, NS, Canada; ^2^Computer Science, King Khalid University, Abha, Saudi Arabia

**Keywords:** persuasive strategies, mental health, mobile application, evaluation, implementation

## Abstract

Mobile applications have shown promise in supporting people with mental health issues to adopt healthy lifestyles using various persuasive strategies. However, the extent to which mental health apps successfully employ various persuasive strategies remains unknown. Hence, it is important to understand the persuasive strategies integrated into mental health applications (apps) and how they are implemented to promote mental health. This paper aims to achieve three main objectives. First, we review 103 mental health apps and identify distinct persuasive strategies incorporated in them using the Persuasive Systems Design (PSD) model and Behavior Change Techniques (BCTs). We further classify the persuasive strategies based on the type of mental health issues the app is focused on. Second, we reveal the various ways that the persuasive strategies are implemented/operationalized in mental health apps to achieve their intended objectives. Third, we examine the relationship between apps effectiveness (measured by user ratings) and the persuasive strategies employed. To achieve this, two researchers independently downloaded and used all identified apps to identify the persuasive strategies using the PSD model and BCTs. Next, they also examine the various ways that these strategies are implemented in mental health apps. The results show that the apps employed 26 distinct persuasive strategies and a range of 1–10 strategies per app. *Self-monitoring* (*n* = 59), *personalization* (*n* = 55), and *reminder* (*n* = 49) were the most frequently employed strategies. We also found that anxiety, stress, depression, and general mental health issues were the common mental health issues targeted by the apps. Finally, we offer some design recommendations for designing mental health apps based on our findings.

## Introduction

Nowadays, mental health issues have become a major public health challenge. People with mental health issues find it difficult achieving their daily tasks such as work and study (Keyes, 2005). As result, many of them are using digital applications to support their mental health and enhance life quality. More than 10,000 mental health and wellness apps are available for download and use (Torous and Roberts, 2017) online. The ubiquitous nature of smartphones and other handheld mobile devices are shaping-up users' lifestyles by adding new aspects to the concept of socializing, accomplishing actions, and creating new habits (Oulasvirta et al., 2012). Therefore, smartphones are attractive platforms for researchers to deliver interventions. Mobile applications (apps) are being used to deliver interventions targeting various health issues (Iacoviello et al., 2017). For mental health issues specifically, Roepke et al. (2015) and Arean et al. (2016) highlighted in their studies that mobile-based mental health intervention made a strong impact on reducing depressed mood. However, they also reported a high rate of drop-out.

By applying various persuasive strategies to reinforce, change, or shape users' behavior and/or attitudes, mental health apps can effectively function as support tools that also motivate and stimulate users to keep on using the apps to achieve better mental health. However, the extent to which available mental health apps successfully employed persuasive strategies and how they implement them in their app to achieve their intended objective remains unknown.

Therefore, this paper aims to achieve three main objectives. First, we review 103 mental health applications and identify distinct persuasive strategies incorporated in them using the Persuasive Systems Design (PSD) model and Behavior Change Techniques (BCTs). We further classify the persuasive strategies based on the type of mental health issues the app is focused on. Second, we reveal the various ways that the persuasive strategies are implemented/operationalized in mental health applications to achieve their intended objectives. Third, we examine whether there is relationship between apps effectiveness (measured by user ratings) and the persuasive strategies employed. To achieve this, two researchers independently downloaded and used all identified apps to identify the persuasive strategies using the PSD model and BCTs. Next, they also examine the various ways that these strategies were implemented in the mental health apps. The results show that the apps employed 26 distinct persuasive strategies and a range of 1–10 strategies per app. Self-monitoring (*n* = 59), personalization (*n* = 55), and reminder (*n* = 49) were the most frequently employed. We also found that anxiety, stress, depression, and general mental health issues were the common mental health issues targeted by the apps. Finally, we offer some design recommendations for designing mental health apps based on our results.

Identifying the persuasive strategies in mental health apps, classifying them based on the type of mental health issues the apps target, and uncovering the relationship between app effectiveness and persuasive strategies employed would be valuable for both researchers and developers working in the mental health domain to inform the design of mental health apps.

## Background

Interactive systems that are designed to change users' behavior or attitude in an intended way are called Persuasive Systems (PSs) (Fogg, 2009). Persuasive systems are widely used in the health and wellness domains to encourage and help users to change their behaviors and/or attitudes.

According to Fogg's Behavior Model (FBM) (Fogg, 2009), there are three factors that help users to perform their target behavior and/or attitudes. These factors are motivation, ability, and triggers. Increasing these three factors is the main focus of persuasive systems. The aim of FBM is to assist researchers and designers to think more about the target behavior that needs to be changed and understand how to design persuasive systems to achieve the desired outcome (Fogg, 2009).

Over the years, many frameworks and taxonomies exist to help designers of persuasive systems in understanding and deconstructing techniques employed in persuasive systems design. Harjumaa and Oinas-Kukkonen (2009) proposed 28 principles of persuasive system design (PSD) model based on three stages of PS development: (1) understanding the main issue behind PS, (2) analyzing the context of PS, and (3) describing different methods to design system features. These principles were classified into four main categories: primary task support, dialogue support, credibility support, and social support categories. Similarly, Abraham and Michie (2008) developed the Behavior Change Techniques (BCTs) which consists of 26 BCTs taxonomies. This Taxonomy was extended later by Michie et al. (2013) to include 93 BCTs, called Behavior Change Technique Taxonomy.

Many researchers used either the PSD model or BCTs or a combination of both to study and deconstruct the persuasive strategies employed in persuasive systems in many areas including web-based health interventions (Kelders et al., 2012) and mobile health interventions (Almutari and Orji, 2019).

Several studies include conducting a systematic review to identify persuasive strategies implemented in health applications using PSD/BCT. For example, Kelders et al. (2012) conducted a systematic review of web-based health interventions and used PSD model to identify which persuasive strategies were most commonly employed and how they affected adherence to the interventions. Their results show that most web-based persuasive systems employed strategies from the primary task support categories including tunneling, tailoring, reduction, and self-monitoring compared to the strategies in the social support category. However, while social support strategies were less commonly employed in web-based interventions, they show a significant contribution to better adherence. In contrast, primary task support strategies that were mostly implemented in web-based interventions did not show any predictive value for adherence. Kelders et al. stated that using persuasive strategies can demonstrate a significant amount of difference in adherence.

Lehto and Oinas-Kukkonen (2011) focused on identifying the persuasive strategies in web-based alcohol and smoking interventions using PSD model. They found that primary task support strategies such as reduction and self-monitoring were widely employed whereas there was a lack of tailoring, which might mean that the interventions are not targeting a particular audience. Similarly, Crane et al. (2015) reviewed popular alcohol-related apps to identify BCTs and discovered that facilitating self-recording information on the consequences of excessive alcohol use, alongside performance feedback, were these apps' most employed strategies.

Matthews et al. (2016) conducted a systematic review of 20 research papers to describe the use of persuasive strategies on mobile apps promoting physical activity using the PSD model. They found that self-monitoring was the most commonly employed strategy whereas system credibility support category was absent in most reviewed mobile applications. However, credibility support strategies, including surface credibility and expertise, were the highest implemented strategies for chronic arthritis apps, followed by general information and self-monitoring (Geuens et al., 2016).

Almutari and Orji (2019) only examined the 32 papers that implemented social support strategies to understand their effectiveness in encouraging physical activity using PSD. They discovered that competition, social comparison, and cooperation are effective strategies to motivate physical activity. For medication management apps for consumers, a reminder was the highest strategy implemented in those apps, followed by tailoring and self-monitoring (Win et al., 2017).

Additionally, Gardner et al. (2016) focused on identifying strategies employed in sedentary behavior reduction interventions using BCT. The study found that the most frequently observed strategies were setting behavioral goals, providing unspecified forms of social support, instruction on how to perform the behavior and self-monitoring. However, self-monitoring, problem solving, and restructuring the social or physical environment were particularly promising behavior-change strategies.

Chang et al. (2012) conducted a systematic review of persuasive strategies in 12 mental health apps to identify the persuasive strategies that are employed in them using the PSD model. They found that primary task support strategies were the most commonly employed whereas social support strategies were least commonly employed. Overall, they concluded that persuasive strategies were not widely employed in mental health applications. Moreover, Wildeboer et al. (2016) examined the relationship between persuasive strategies, adherence, and the effectiveness of web-based intervention for mental health. Results indicated there is a relationship between the number of persuasive strategies and the intervention's effectiveness. We extend existing work by focusing on persuasive strategies in mental health apps and employing both the BCTs and the PSD framework in our review. First, we identify distinct persuasive strategies incorporated in mental health apps and classify the persuasive strategies based on the type of mental health issues the app is focused on. Second, we reveal the various ways that the persuasive strategies are implemented/operationalized in mental health applications to achieve their intended objectives. Third, we examine the relationship between app effectiveness (measured by user ratings) and the persuasive strategies employed. Finally, we offer some design recommendations that help app developers and health professionals to build more effective support tools for people who experience mental health issues.

## Methods

In this section, we describe the methods used to achieve the study objectives. Specifically, we detailed the app selection criteria and the coding.

### Selection of Sample Apps

**Figure 1:**
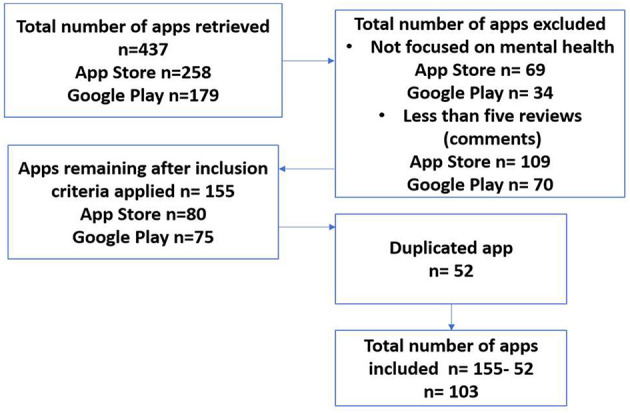
Process of selecting mental health apps.

We searched on the App Store and Google Play using the keywords “mental health,” “anxiety,” “depression,” “mood,” “emotions,” and “stress.” We also searched using various combinations of the keywords joined using the conjunctions “OR” and “AND.” The search result revealed the initial list of 437 apps (258 apps from App Store and 179 apps from Google Play). For our analysis, we included apps whose main goal according to the app's description and the demo of the app show that they are targeted at mental health, and apps that have more than five reviews (comments) in total. In other words, apps that fall into any of these categories are excluded: (1) not focused on mental health, (2) had less than five reviews (or comments), or (3) was not in English. In addition, for apps that appeared in both App Store and Google Play, we counted it as one instead of two. After applying the selection criteria, a total of 103 apps remained and eligible for coding (see Figure 1). The following information was also extracted for each eligible app: *name, platform* (i.e., iPhone, Android, or both)*, developer, date of the last update*, and *price* (i.e., free, fee-based, and free with in-app purchases—where developers provide a free version and a paid version if users want to upgrade or unlock additional features in the app).

### Coding Apps for Persuasive Strategies

The aim of the coding process in our study is to assess the number and type of persuasive strategies present in mental health apps. Collected apps were coded using both the Persuasive System Design model (PSD) (Harjumaa and Oinas-Kukkonen, 2009) by Oinas-Kukkonen and Harjuma, and Behavior Change Techniques BCTs (Michie et al., 2013) by Michie and Abraham. We combined and used both the Oinas-Kukkonen and Michie's frameworks to have a comprehensive list of strategies for deconstructing the apps.

To identify the persuasive strategies employed in the apps and their implementations, a subsample apps (and = 5) were downloaded and used for 10 days by two researchers to ensure there is no new strategy revealed during using. After that two researchers independently downloaded and reviewed the 103 apps to identify the persuasive strategies using the PSD model and BCTs. The researchers then met to agree on the initial codes. For any disagreement that arose between the two researchers, a third researcher was involved to mediate and ensure an agreement is reached. The researchers also classified the persuasive strategies based on the type of mental health issues the app is targeting. Moreover, we also identified the various ways that the persuasive strategies are implemented/operationalized in mental health apps to achieve their intended objectives.

### Analysis

To analyze our data, we employed some well-known analytical approaches:

- First, we measured the percentage of agreement between two researchers (i.e., before the third researcher was involved). We also calculated interrater reliability, kappa and prevalence and bias adjusted kappa (PABAK).
- Second, we conducted descriptive statistics to obtain the mean of persuasive strategies employed in the apps.
- Third, we employed independent-samples *t*-tests to compare the mean of persuasive strategies between free and paid apps and between iPhone and Android apps. Apps that have two versions used on both platforms were not included in the *t*-test analysis.
- Finally, to examine the relationship between the number of persuasive strategies and the effectiveness of apps (as determined by the app ratings), we performed a Pearson's correlation analysis between the number of persuasive strategies and the app's rating.

## Results

We present the detailed results of our analysis in subsequent subsections. We describe the coding agreement, the persuasive strategies employed, their implementations, the target mental health domain, the relationship between the number of strategies employed and app effectiveness.

### Description of Selected Apps

We provide a summary of the app's description in Table 1. Approximately half (47%) of the apps had been updated within the past year (2018). More details of the apps can be found in the Appendix.

**Table 1:** A summary description of 103 mental health apps.

Price	Free (41), fee-based (11%), Free with in app purchases (49%)
Developer	Unknown (16%), Commercial (profit Organization) (69%), Government (7%), NGO (4%), University (5%)
Rating	No rating (7%), 2–2.9 (4%), 3–3.9 (16%), 4–4.9 (69%), 5(4%).
Platform	iPhone (27%), Android (22%), both (50%).


### Persuasive Strategies Employed in Mental Health Apps

**Figure 2:**
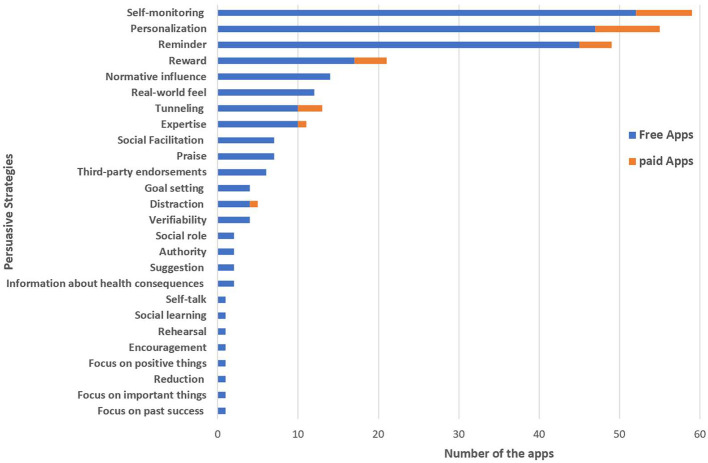
Persuasive strategies employed by mental health apps categorized into free and paid apps.

The results of our analysis show that the percentage of agreement between the two researchers was 86.5%. There was “substantial” agreement: prevalence and bias adjusted kappa (PABAK) = 0.71 (Landis and Koch, 1977; Byrt et al., 1993). Discrepancies were discussed and the coding was refined. Overall, we found 26 distinct persuasive strategies present in the mental health apps reviewed. The number of strategies employed in each app varied and ranges between 1 and 10. However, 14 mental health apps did not employ any persuasive strategies. Interestingly, self-monitoring (*n* = 59), personalization (*n* = 55), and reminder (*n* = 49) emerged as the most commonly employed strategies (see Figure 2). Moreover, we found other strategies that do not exist in PSD/BCT that were employed in the reviewed mental health apps: *Encouragement, focus on positive things* and *focus on important things*.

The results of our *t*-test show that there was a significant difference in the number of persuasive strategies employed within iPhone apps (M = 2.61, SD = 1.524) and Android apps (M = 1.74, SD = 1.137); t(49) = 2.26, *p* = 0.028. These results suggest that the number of persuasive strategies employed within iPhone apps is more than Android apps. However, there was no significant difference in the number of persuasive strategies present in free apps (M = 2.38, SD = 2.073) and paid apps (M = 2.27, SD = 1.104) apps; t(101) = 0.868, *p* = 0.388. These results suggest that the number of persuasive strategies present in free apps is the same in paid apps.

### Persuasive Strategies in Other Health Domains

**Figure 3:**
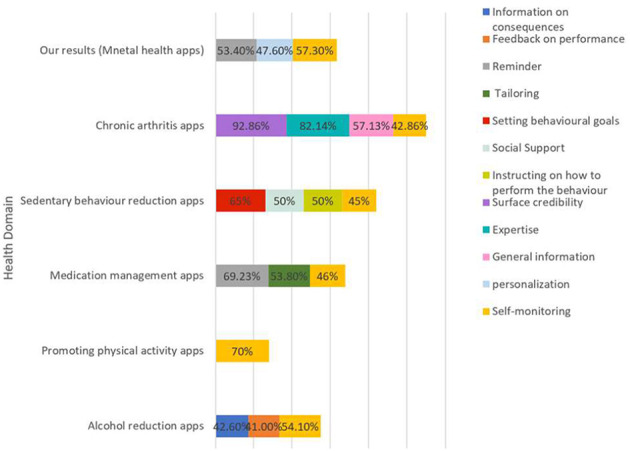
Comparative the most strategies implemented in reviewed apps by other health domains.

Comparison of our findings to the findings of earlier reviewed health care apps studies reveals that self-monitoring is one of the common strategies that emerge in chronic arthritis apps (Geuens et al., 2016), alcohol reduction (Crane et al., 2015), sedentary behavior reduction (Gardner et al., 2016) and promoting physical activities (Matthews et al., 2016). However, Credibility support strategies were the most strategies implemented in chronic arthritis apps (Geuens et al., 2016) whereas those strategies were absent in most reviewed mobile applications for physical activity (Matthews et al., 2016). Moreover, reminders were the most implemented strategy in medication management apps for consumers (Win et al., 2017). Our results revealed that self-monitoring, personalization, and reminder were the most frequently employed strategies in mental health apps. Some of these studies further reported more strategies that might not frequently be implemented in other health care apps (see Table 2 and Figure 3).

**Table 2:** Most strategies implemented in reviewed apps in other health domains.

**References**	**Health domain**	**model used**	**Most strategies implemented**
Crane et al. (2015)	Alcohol reduction	BCTs	• Self-recording (self-monitoring) • Information on consequences • Feedback on performance
Matthews et al. (2016)	Promoting physical activity	PSD	• Self-monitoring
Win et al. (2017)	Medication management	PSD	• Reminder • Tailoring • Self-monitoring
Gardner et al. (2016)	Sedentary behavior reduction	BCTs	• Setting behavioral goals • Social support • Instruction on how to perform the behavior • Self-monitoring
Geuens et al. (2016)	Chronic arthritis	BCTs and PSD	• Surface credibility • Expertise • General information • Self-monitoring
Our results	Mental health apps	BCTs and PSD	• Self-monitoring • Personalization • Reminder

### Persuasive Strategies and Type of Mental Health Issues Targeted

**Figure 4:**
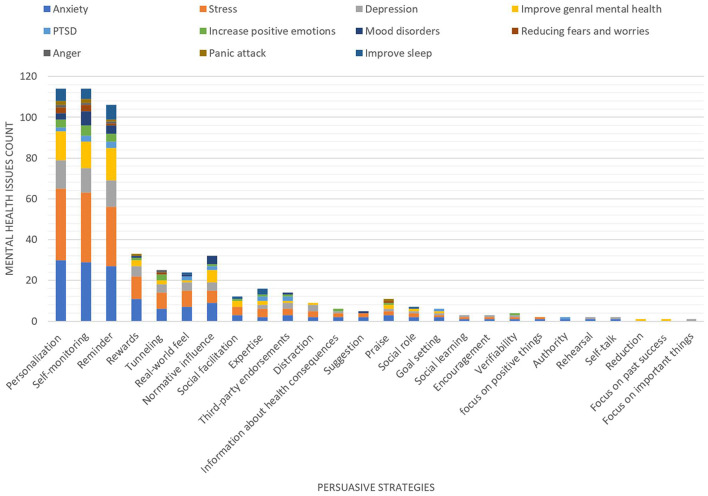
Persuasive strategies for each mental health issue.

**Figure 5:**
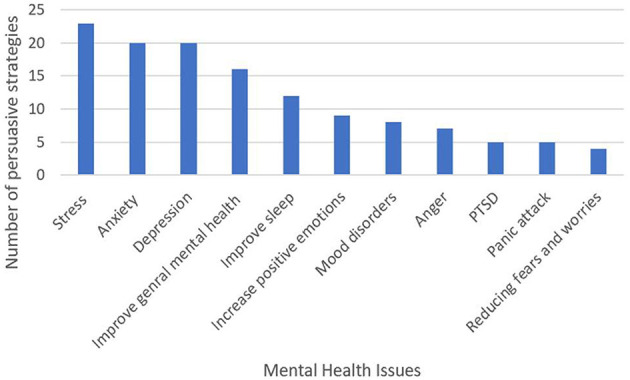
Number of persuasive strategies employed in each mental health issues.

We examined the persuasive strategies and the type of mental health issues the apps target. The results show that 65 apps target a combination of mental health issues whereas only 38 apps target a specific mental health issue. In general, the apps mostly targeted the following mental health issues: *anxiety, stress, depression*, and *general mental health* (see Figure 4). However, apps that targeted *stress* employed the highest number of persuasive strategies (23 out of 26 persuasive strategies identified in all mental health apps), followed by apps targeting *anxiety* and *depression* employed 20 persuasive strategies. Figure 5 presents the overall number of persuasive strategies employed in each mental health issues. The results also show that personalization, self-monitoring and reminder were the most employed persuasive strategies in various mental health apps, see Figure 3.

### Implementation of Persuasive Strategies

We present the various implementation of the common persuasive strategies in mental health application in this section.

#### Self-Monitoring Strategy

Self-monitoring strategy “allows people to track their own behaviors, providing information on both past and current” (Orji et al., 2017b, 2018c). In 23 apps, *self-monitoring* was implemented as being able to review trends of personal data related to mental health in a calendar or graphical format. Moreover, another 29 apps offer *self-monitoring* as a total number of activities related to mental health improvement performed by an individual and how long they spent on each activity. These include activities such as meditations.

#### Personalization

Personalization offers tailored contents, functionalities, and services to suit user's needs and choices. Tailoring content and functionality to a particular user's need based on his/her characteristics increases the efficacy of the system (Orji et al., 2017b, 2018c). Personalization was implemented in mental health apps in various ways including customizing the appearance of the app (such as background, theme, and sounds) to an individual's preference, customizing some app's functionalities (such as breathing rate, meditation duration, and music duration), providing some functionalities that allow individuals to adapt the apps to suit their personal preference (such as music, picture, activities, and challenges), tailoring the content based on certain user's characteristics (such as adapting meditation based on user's current emotion state).

#### Reminder Strategies

Reminder strategies enables a system to remind user to perform the target behavior. *Reminder* emerged as one of the popular strategies used in mental health apps. It is implemented in 49 mental health apps mainly to remind users to perform an activity (such as meditation, breathing, and assessment) or to track their personal data (e.g., mood). *Reminders* are often implemented as alert or pop-up boxes and sound.

#### Reward and Praise Strategies

*Reward* “offers virtual rewards to users for performing the target behavior” (Orji et al., 2017b) while **Praise** “applauds the user for performing the target behavior via words, images, symbols, or sounds as a way to give positive feedback to the user” (Orji et al., 2014). Mental health apps provide *reward* in various forms. Some mental health apps provide *reward* in form of points (6 apps), badges (8 apps), trophies (1 app), insight sticker (1 app), coins (1 app), planet growth (2 apps), and streak (1 app) that could be collected or gained while completing a task such as breathing and meditation. Only 2 apps implemented reward by allowing users to unlock more contents (such as more meditation sessions, or more lessons and activities) as a way of rewarding users. With respect to the Praise strategy, only 7 apps employed praise as words (i.e., Well Done) (6 apps) and colorful confetti (1 app).

#### Normative Influence Strategy

Normative influence strategy allows a system to provide “means for gathering together people who have the same goal and make them feel norms” (Harjumaa and Oinas-Kukkonen, 2009). *Normative influen*ce was implemented as a means for social interaction in form of community forums (13 apps) where users can exchange views about issues and feelings or by providing a link to join a Facebook community group (1 app).

#### Social Facilitation Strategy

Social facilitation strategy allows the system to provide a means for discerning other people who are performing the target behavior (Harjumaa and Oinas-Kukkonen, 2009). *Social facilitation* was implemented in the form of community forum of follower and following or listener and listening. Connected people can see each other's activities; followers can see the activities of the people they are following.

#### Credibility Strategy

Credibility strategy posits that systems should have competent look and feel (Harjumaa and Oinas-Kukkonen, 2009) to attract users and motivate behavior. The reviewed mental health apps employed many approaches to influence system credibility such as displaying **Expertise** (i.e., expertise in the design of the components of the app and knowledgeable in the information provided) (11 apps), providing **Real-world feel** [i.e., highlighting the people behind the design of the app (12 apps)], and by showcasing **Authority** [i.e., referencing notable organizations who are authority in the area of mental health (2 apps)]. Moreover, **Third-party endorsements** were employed in only 6 apps by providing a logo of respected sources such as a logo of a known university that is behind the app or approved the app. Moreover, few mental health apps implemented **Verifiability** (4 apps), allowing users to find more information by linking to studies or reports that provide evidence to support their claim or evidence that informed their design.

#### Tunneling Strategies

Tunneling strategies posits that system should guide users through the step-by-step process that lead to the target behavior by providing means for action that brings them closer to the target behavior. *Tunneling* was implemented in 13 mental health apps in the form of guidance on how to use the app to achieve a specific activity in line with the desired mental health outcome (e.g., how to meditate and how to breathe properly).

#### Encouragement

Encouragement was designed as a supportive message, and positive motivational quotes.

#### Focus on Positive Things

Focus on positive things was designed, as a game intended to help users learn how to focus on positive words or events as a way of promoting mental health and decreasing negativity.

#### Focus on Important Things

Focus on important things was implemented as a game intended to teach users how to focus on important things in users' life and avoid distraction and cognitive overload as a way of promoting mental health.

#### Distraction

Distraction was also implemented as a simple game aimed at diverting user's attention and distracting them from the current (negative) mood.

### App Effectiveness and Persuasive Strategies Employed

To examine whether there is a relationship between the number of persuasive strategies employed in the app design and the perceived app effectiveness (as assessed by the app ratings), we performed Pearson's correlation between the app rating and the number of persuasive strategies. The results show that there is no relationship between the number of persuasive strategies and the app effectiveness; *r* = 0.153 (no correlation), *n* = 103, and *p* = 0.123. So, there is no significant correlation between the number of persuasive strategies and app rating, which demonstrates the perceived effectiveness from the user's point of view.

## Discussion

In this Section, we discuss our findings and offer some design recommendations for mental health app based on our findings.

### Persuasive Strategies and Implementation

The purpose of this study is to identify distinct persuasive strategies incorporated in mobile apps designed to improve mental health and classify the persuasive strategies based on the type of mental health issues the app is focused on. Moreover, the study aims to reveal the various ways persuasive strategies are implemented/operationalized in mental health applications to achieve their intended objectives, and also to examine the relationship between apps effectiveness and the persuasive strategies employed.

Overall, the mental health apps reviewed in this paper employed 26 persuasive strategies, a range of 1 to 10 per app. However, 14% of the mental health apps did not employ any persuasive strategies.

Unsurprisingly, we found that self-monitoring is the most prevalent persuasive strategies implemented in mental health apps. According to Bakker et al. (2016), self-monitoring is considered the main feature of many evidence-based psychological therapeutic techniques such as cognitive behavior therapy, mindfulness exercises, emotion-focused therapy, Dialectical behavior therapy (DBT), and acceptance and commitment therapy (ACT). Self-monitoring help users with mental health issues to manage their conditions. They can track their feeling, thoughts, and behaviors which in turn increases self-awareness and improve mental health outcomes. The apps reviewed in this study limited tracking to manual input. Users with mental health issues need to enter their personal data manually which indeed is a major limitation. As highlighted by Orji et al. (2018a), manual recording is tedious, time consuming, and may not work for people with serious mental health issues.

Personalization emerged as the second most employed persuasive strategies in mental health apps. According to Price et al. (2016), personalizing some aspects of an app such as changing colors, setting backgrounds, and personalizing assessment questions would improve an app's usability. Most importantly, the ability to adjust the intervention delivered via mental health apps to suit the user's needs and characteristics will make the intervention more effective (Orji et al., 2014). Moreover, it has been found that personalized health interventions are more effective than the ones employing the one-size-fits all approach in other health domains (Orji, 2014; Orji et al., 2017a) and in depressive and anxiety disorders specifically (Carlbring et al., 2011; Silfvernagel et al., 2012). Therefore, the fact that most mental health app incorporated some form of personalization is not surprising considering that even people suffering from the same or similar mental health conditions may have unique needs that require individualized solutions.

Although tunneling and reduction reduces the effort required to achieve the target behavior by guiding the users to perform the task (tunneling) and by simplifying the task (reduction), it was incorporated only in 17 apps and 1 app, respectively. This is surprising, considering that individuals experiencing mental health conditions are often required to avoid stressful situations including complicated tasks that may stress them out and worsen their situation. Hence, making it essential that mental health apps are simple enough and also can guide users through the step-by-step process required to achieve the desired behavior is necessary to reduce the tendency of stressing them out when figuring things out themselves. Moreover, according to Alqahtani and Orji (2019), users with mental health issues complained about lack of guidance when using mental health apps which impair concentration and make them be easily frustrated. Therefore, reduction and tunneling strategy are essential for mental health apps.

The third persuasive strategy present in mental health applications is the reminder strategy. This strategy is mostly designed to remind users to track their personal data or to perform some mental health improvement activities such as meditation and breathing. Although reminders can help to increase adherence and reduce dropout from intervention, it was only implemented in 49 apps out of 103 apps. However, according to Bakker et al. (2016), a lot of annoying reminders can lead to disengagement. Therefore, developers should be careful when designing the reminder in mental health apps to avoid annoying people with frequent and unsolicited reminders. One way to achieve a balance between providing an effective reminder that will encourage users to adhere to the intervention and avoiding unnecessary reminders that will annoy user and make them disengage from the app is to tailor reminders to each individual. Individuals can be allowed to customize not only the frequency at which reminders are sent to them (how often), but also the type of reminder (pop up boxes, text message, sounds etc.) and when it should be sent (time).

Moreover, persuasive strategies such as reward and praise have been found to be among the popular strategies employed in many health apps to motivate users to be more engaged (Orji et al., 2014). However, only a few mental health apps implemented reward and praise, although apps for users with mental health issues may benefit from these techniques to motivate users. The reason why reward and praise are not as popular strategies in mental health apps is probably because many designers believe that improving mental health is an intrinsic reward of using their app hence no extra reward is required. Although, improving mental health is a major benefit and the main reason why many people would resort to using the app in the first place, it does not overwrite the need for extrinsic rewards such as badges and points, which have been shown to be effective at engaging users (Orji et al., 2013). According to Orji et al. (2018b), performing health behaviors is often difficult due to lack of immediate tangible benefit, offering intermediate rewards such as points and badges, may help to engage the users while the await the intrinsic reward.

The first credibility strategy is *real-world feel* and it is characterized by providing information about people or organization behind the app's content. This was found in 12 apps and still surprisingly low. In addition, 11 apps offered expertise in the design of the components of the app and information provided. We argue that these strategies are very important. All mental health apps, like all health apps, should provide information that is scientifically proven and evidence-based. Possessing the adequate technical skills to be able to develop an app is not enough for designing apps that will effectively improve or support mental health. Unfortunately, only 4 apps implemented the verifiability strategy which offers a way for users to verify the apps' content, 6 apps employed the third-party endorsements and 2 apps employed the authority which were very low. Credibility strategies are very important in mental health applications considering the sensitivity of the subject matter. Users need to be assured of not only the effectiveness and reliability of the app contents, but also that their data will be protected (privacy). In the Google store and App stores, anyone can design an app and publish it without providing evidence on the effectiveness of methods used in the app to manage the mental health issues and how users' information is protected. This results in a substantial number of apps that are not perceived as credible and trustworthy.

Social support is an important strategy for users who experience mental health issues because most of them often feel isolated or stigmatized. In this review, we found that only a few apps employed the strategies in the social support category. Only 17 apps employed the normative influence strategy which allow users to share their issues, thoughts, emotions with others to find support.

Certain strategies revealed in our study liken those identified in reviewed applications of other health domains. For example, self-monitoring strategies were similarly highlighted in reviewed applications of medication management apps for consumers (Win et al., 2017), chronic arthritis apps (Geuens et al., 2016), and promoting physical activities (Matthews et al., 2016). Moreover, self-monitoring strategies were specifically highlighted as a promising approach to sedentary behavior reduction (Gardner et al., 2016). However, it is worth mentioning that there are other strategies that emerge as most employed in the reviewed applications in other health domains that are not in mental health applications such as credibility support (Geuens et al., 2016).

In general, only a few persuasive strategies were employed in the apps that we reviewed which might explain the high attrition rate.

### Persuasive Strategies and Type of Mental Health Issues

Most of the mental health apps that we reviewed targeted a combination of mental health issues which make it hard to know which persuasive strategies are more effective for a specific mental health issue. However, personalization, self-monitoring and reminder remain the most employed persuasive strategies in various mental health issues. Anxiety, stress, depression, and general mental health issues were the most issues the apps in this review target.

### Apps Effectiveness and Persuasive Strategies Employed

The effectiveness of the app was measured based in the app's rating. Interestingly, we found no relationship between the number of persuasive strategies and apps effectiveness as indicated by users' ratings. This is particularly an interesting result considering the recent discussion and open research question on whether persuasive systems employing a multiple persuasive strategy are more effective than those employing a single strategy (Orji et al., 2017a). Our findings suggest that the number of strategies employed in apps design may not be related to the apps' effectiveness. According to Orji et al. (2017a), this is probably because employing a single appropriate strategy may be better than employing multiple inappropriate strategies or a combination of appropriate and inappropriate strategies that may have a cancellation effect. Hence, it is important that designers focus on selecting the appropriate persuasive strategies having both the target audience and the target behavior in mind.

### Design Recommendation

Based on our findings, in this section, we offer some recommendations for designing mental health applications to improve users' adherence and engagement and hence apps' effectiveness. Moreover, some recommendations provided are from user app reviews, although the qualitative comments are not the focus of this work, we have integrated certain comments to support our recommendations.

1- Designer should employ self-monitoring in the apps that target mental health issues to help users to track their personal data and see their improvements over time. Allowing people with mental health issues to track and visualize their personal data in various format, would provide opportunity for self-awareness and help users take control of their mental health management. For example, “I love the app. It allows you to track emotions, experiences, discoveries, actions you took” [R29]. A major drawback is that most mental health app that employ self-monitoring use manual tracking which makes them tedious, time consuming, and users are likely to forget. To overcome this limitation, we suggest that designers employing self-monitoring should simplify the process and reduce the amount of work involved by automating behavior monitoring process whenever possible (Orji et al., 2018a) Although certain behavior data cannot be automatically tracked without users' involvements due to technology limitation. Therefore, for such behaviors that cannot be automatically monitored, designers should incentivize users, and reduce the perceived tediousness of the self-monitoring process using complementary persuasive strategies such as reminding users to log their behavior, rewarding users for tracking their behaviors each day, and reducing the number of steps required to record behavior” (Orji et al., 2018a).
2- Provide adaptive functionalities that allow users to adapt some app features such as the font size, font color, background, layout, type and length of meditation, breathing, and other mental health improvement tasks to suit each user's preferences and unique mental health needs. *For example, “The breathing exercises are great because I can set the type and time which I see as a great feature” [R487]*. Personalization increases system relevance and usefulness (Orji et al., 2017c), enhances system's overall usability and ensure a personalized experience for each user. Moreover, adjusting app contents based on user's personal data will increase the effectiveness of the mental health interventions. In addition, since many mental health applications targets more than one mental health issues, it is necessary that the apps' content be adapted based on the type of mental health issues that users might be experiencing. However, even people suffering from the same or similar mental health conditions may have unique needs that require individualized solutions, hence highlighting the need to personalize mental health apps to each individual.
3- Provide an adequate reminder to remind user to track their data or to perform their meditation, breathing, and other mental health-related task. *For example, “I like a short notification “How are you feeling?” from time to time” [R213]*. Although reminders can help to increase adherence and reduce dropout rate, a lot of annoying reminders can lead to disengagement. Therefore, developers should be careful when designing the reminder in mental health apps to avoid annoying people with frequent and unsolicited reminders. One way to achieve a balance between providing an effective reminder that will encourage users to adhere to the intervention and avoiding unnecessary reminders that will annoy user and make them disengage from the app is to tailor reminders to each individual. Individuals can be allowed to customize not only the frequency at which reminders are sent to them (how often), but also the type of reminder (pop up, text message, sounds etc.) and when it should be sent (time).
4- The mental health apps should provide a means for users to verify the reliability of their content and provide mental health information that are scientifically proven and endorsed by expert third parties. *For instance, “It works, and the science behind it is impressive” [R7*]. This will increase app credibility hence motivating users with mental health issues to engage with the intervention (Bakker et al., 2016). Possessing the adequate technical skills to be able to develop an app is not enough for designing apps that will effectively improve or support mental health. Credibility strategies are particularly important in mental health applications considering the sensitivity of the subject matter. Users need to be assured of not only the effectiveness and reliability of the app contents, but also that their data will be protected (privacy).
5- Mental health apps could benefit from implementing rewards and praise. For example, “*the growing tree is a nice way to see my practice is growing*” [R201]. Showing the growing tree as a kind of reward might motivate users to engage with the app. Moreover, providing users with mental health with motivational message when finishing the activities or task might encourage them continue using the app. Although designers may argue that improving mental health is an intrinsic reward of using their app hence no extra reward is required, however, it does not overwrite the need for extrinsic rewards such as badges, points, which has been shown to be effective at engaging users (Orji et al., 2013). According to Orji et al. (2018b), performing health behaviors is often difficult due to lack of immediate tangible benefit, offering intermediate rewards such as points, badges, may help engage the users while the await the intrinsic reward.
6- Employ Reduction and Tunneling to simplify mental health apps and guide users through the step-by-step process required to achieve the desired mental health outcome. *For example, “Users of the app are guided step by step in using every aspect to support their emotional health” [R73]*. This will also reduce the tendency of stressing users out by allowing to figure things out themselves and hence reduce the overall dropout rate. Individuals experiencing mental health conditions are often advised to avoid stressful situations including complicated tasks that may stress them out and worsen their situation.
7- Employ the Social Support strategies in mental health apps (e.g., user forums) to provide users opportunity share their experience and support each other. Most people suffering from mental health issues often feel isolated or stigmatized, hence the need for social support. *For example, “It's a really good way to connect and feel connected to other people who have the same problem as you; even if you think you're alone” [R73]*.

## Limitations

This study has several limitations. Firstly, there exists the possibility that we missed some strategies due to the short timeframe of subsample applications. One method for overcoming this limitation is to extensively use a subsample for a longer duration to ensure no additional persuasive strategies are unrevealed. Secondly, user ratings are not enough to measure the effectiveness of apps because many other factors can affect the effectiveness of apps. However, user rating was the singular, closest evaluation we had to measure effectiveness.

## Conclusion

In this paper, we deconstructed distinct persuasive strategies employed in 103 mental health applications using the Persuasive Systems Design (PSD) model and Behavior Change Techniques (BCTs). Two researchers independently coded 103 apps descriptions using the PSD model and BCTs. We further classified the persuasive strategies based on the type of mental health issues the apps aimed to address and how the strategies are implemented/operationalized in the mental health apps. The results show that self-monitoring, personalization, and reminder are the most commonly employed persuasive strategies in mental health apps irrespective of the mental health issues. We also found that anxiety, stress, depression, and general mental health are the mental health issues the apps mostly focused on. Above all, we uncovered that there is no relationship between the number of persuasive strategies employed and apps' effectiveness as measured using user ratings. We discuss various ways each persuasive strategy was implemented in mental health app to achieve the desired objective. Finally, we offered some design recommendations for mental health apps based on our findings. Future study should investigate which persuasive strategies are deemed as more important by users with mental health issues. We also hope to apply our recommendations in designing and evaluating mental health apps.

## Data Availability Statement

All datasets generated for this study are included in the article/Supplementary Material.

## Author Contributions

FA designed the study. FA, OO, and GA collected the data. FA analyzed the data and wrote the manuscript. RO and OO reviewed the manuscript. RO supervised the study.

### Conflict of Interest

The authors declare that the research was conducted in the absence of any commercial or financial relationships that could be construed as a potential conflict of interest.
