# Supplementary material for: Apps for Mental Health: An Evaluation of Behavior Change Strategies and Recommendations for Future Development
Source: Front Artif Intell. 2019 Dec 17;2:30. doi: 10.3389/frai.2019.00030 (PMC7861234; doi:10.3389/frai.2019.00030)
Supplement: Supplementary file 1 [file Data_Sheet_1.docx]

Appendix 1: Details and persuasive strategies of Mental Health Apps

| S/N | App Name | Rating | platform | price | developer | Category | Last update | Persuasive Strategies | Mental Health Type App focuses on |
| --- | --- | --- | --- | --- | --- | --- | --- | --- | --- |
| 1 | CPT Coach | No Rating | Both | Free | Government | Health &fitness | August 31, 2017 | Reminder Self-monitoring Third-party endorsements Expertise Real-world feel | Post-traumatic stress disorder (ptsd) |
| 2 | MindShift | 3.1 | Both | Free | NGO | Health &fitness | August 24, 2016 | Personalization Self-monitoring Goal setting  Reminder | Anxiety |
| 3 | Pacifica | 4.7 | Both | Free * | Commercial  (Profit Organizations) | Health &fitness | March 9, 2018 | Self-monitoring  Normative influence  suggestion  Personalization  Reminder | Anxiety, mood disorders and stress, |
| 4 | T2 Mood Tracker | 3.8 | Both | Free | Government | Health &fitness | October 11, 2016 | Reminder  Third-party endorsements  Self-monitoring  Personalization | anxiety, stress, depression, post-traumatic stress, general well-being |
| 5 | PTSD Coach | 4.2 | Both | Free | Government | Health &fitness | August 1, 2017 | Real-world feel  Self-monitoring  Reminder  Personalization  Authority  Goal setting  Expertise | posttraumatic stress disorder (PTSD) |
| 6 | WhatsMyM3 | 5 | Both | Free | Commercial  (Profit Organizations) | Medical | May 31, 2016 | Self-monitoring | Mood disorder |
| 7 | Self-help for Anxiety Management (SAM) | 3.5 | Both | Free | University | Health &fitness | February 21, 2017 | Self-monitoring  Real-world feel  Normative influence  Authority  Personalization | anxiety |
| 8 | Anxiety Reliever: Meditation | 3 | Both | Free * | Commercial  (Profit Organizations) | Health &fitness | November 27, 2016 | Self-monitoring | anxiety and stress |
| 9 | MoodTools | 3.8 | Both | Free | Unknown | Medical | May 19, 2017 | Tunneling  Reminder  information about health consequences  Personalization  Self-monitoring | Reduce negative emotions, Depression, Anxiety/Stress |
| 10 | Breathe2Relax ) | 4.5 | Both | Free | Government | Health &fitness | June 6, 2016 | information about health consequences  Personalization  Third-party endorsements  Self-monitoring | Anxiety/Stress |
| 11 | Happify | 4.5 | Both | Free * | Commercial  (Profit Organizations) | Health &fitness | March 19, 2018 | Normative influence  Praise  Self-monitoring  Tunneling  Third-party endorsements  Reminder  Social facilitate  Verifiability  Personalization  Reward | increase positive emotions |
| 12 | Headspace | 4.9 | Both | Free * | Commercial  (Profit Organizations) | Health &fitness | March 12, 2018 | Self-monitoring  Reminder  Personalization  Social facilitation  rewards | anxiety and stress |
| 13 | Calm | 4.8 | Both | Free * | Commercial  (Profit Organizations) | Health &fitness | February 26, 2018 | Personalization  Self-monitoring  Reminder | anxiety, improve sleep, and help to feel happier |
| 14 | Moodpath | 4.6 | Both | Free * | Commercial  (Profit Organizations) | Medical | March 19, 2018 | Self-monitoring  Reminder  Personalization | Depression, Anxiety/Stress |
| 15 | SuperBetter | 4.7 | Both | Free | Commercial  (Profit Organizations) | Health &fitness | March 1, 2018 | Personalization  Social learning  Reward  Reminder  Real-world feel | Depression, Anxiety/Stress, |
| 16 | 7 Cups | 2.4 | Both | Free * | Commercial  (Profit Organizations) | Health &fitness | March 20, 2018 | Normative influence  rewards  Reminder | anxiety and depression. |
| 17 | BoosterBuddy | 4.6 | Both | Free | Government | Health &fitness | December 18, 2017 | Self-monitoring.  Reminder  Personalization  Reward | general mental health |
| 18 | Relax Lite: Stress and Anxiety Relief | 4.7 | Both | Free * | Commercial  (Profit Organizations) | Health &fitness | August 7, 2017 | Personalization  Rewards  Self-monitoring | Anxiety/Stress |
| 19 | Aura | 4.9 | Both | Free * | Commercial  (Profit Organizations) | Health &fitness | March 22, 2018 | Self-monitoring  Personalization  Social facilitation  suggestion  rewards  Normative influence  Reminder  Praise | stress & anxiety |
| 20 | CBT Thought Record Diary | 4.6 | Both | Free * | Unknown | Medical | June 5, 2017 | Self-monitoring | Anxiety/Stress |
| 21 | Smiling Mind | 5 | Both | Free | NGO | Health &fitness | August 1, 2017 | Reminder  Self-monitoring  Tunneling  Real-world feel  Expertise  Personalization | stress and challenges of daily life. |
| 22 | What's Up? - A Mental Health App | 4.8 | Both | Free | Unknown | Health &fitness | December 5, 2016 | Personalization  Self-monitoring  Goal setting  distraction  Real-world feel  Praise  Encouragement  Reward  Normative influence | Depression, Anxiety, Anger, Stress |
| 23 | IMoodJournal | 4.7 | Both | Paid | Commercial  (Profit Organizations) | Lifestyle | March 14, 2018 | Self-monitoring  Personalization  Reminder | mood, sleep, stress and anxiety, energy level, |
| 24 | ACT Companion: Happiness Trap | 4.2 | Both | Free * | Commercial  (Profit Organizations) | Health &fitness | December 6, 2017 | Self-monitoring  Personalization  Expertise  Verifiability  Reminder | anxiety and depression as well as mental wellbeing |
| 25 | MoodMission | 4 | Both | Free * | Commercial  (Profit Organizations) | Health &fitness | January 10, 2018 | Self-monitoring  Third-party endorsements  Real-world feel  Reward | Depression, Anxiety/Stress, mood disorders |
| 26 | Virtual Hope Box | 4 | Both | Free | Government | Health &fitness | October 11, 2016 | Personalization  Third-party endorsements  distraction | Depression |
| 27 | Thought Challenger | No Rating | Both | Free | University | Health &fitness | October 9, 2017 | Nothing | negative emotions |
| 28 | Tactical Breather | No Rating | Both | Free | Government | Health &fitness | December 20, 2014 | Tunneling  Personalization  Verifiability | Anxiety/Stress |
| 29 | End Anxiety Hypnosis - Stress, Panic Attack Help) | 4.6 | Both | Free * | Commercial  (Profit Organizations) | Medical | October 15, 2017 | Nothing | anxiety |
| 30 | Relax with Andrew Johnson | No Rating | Both | Paid | Unknown | Medical | January 8, 2018 | Reminder  Personalization | stress/anxiety |
| 31 | At Ease: Anxiety & Worry Relief | 5 | Both | Paid | Commercial  (Profit Organizations) | Health &fitness | August 13, 2017 | Tunneling  Personalization | Anxiety/Stress |
| 32 | Cure Stress - unique technique for relief of anxiety, pain, stress, insomnia and more | 4.8 | Both | Free * | NGO | Health &fitness | November 2, 2014 | Reminder  Real-world feel | Anxiety/Stress, reduce negative emotion/ fear/ pain |
| 33 | Guided Mind | 4.6 | Both | Free * | Commercial  (Profit Organizations) | Health &fitness | December 19, 2017 | Personalization | stresses and challenges of day-to-day life. |
| 34 | Wildflowers Mindfulness | 4.5 | Both | Free | Commercial  (Profit Organizations) | Health &fitness | November 7, 2017 | Personalization  Reward  Reminder | general mental health |
| 35 | HelloMind | 3.7 | Both | Free * | Commercial  (Profit Organizations) | Health &fitness | March 29, 2018 | Nothing | stress, and bad sleep, |
| 36 | Omvana - Meditation for All | 4 | Both | Free * | Commercial  (Profit Organizations) | Health &fitness | October 3, 2017 | Personalization  Reminder | Anxiety/Stress, |
| 37 | Relieve Depression Hypnosis | 4 | Both | Free * | Commercial  (Profit Organizations) | Health &fitness | September 26, 2018 | Personlization | depression |
| 38 | Insight Timer-Meditation App | 4.8 | Both | Free * | Commercial  (Profit Organizations) | Health &fitness | January 14, 2019 | Self-monitoring  Social facilitation  Real-world feel  Normative influence  Personalization  Reminder | Anxiety and Stress |
| 39 | AnxietyCoach | 3.2 | iPhone | Paid | NGO | Health &fitness | October 31, 2017 | Personalization  Self-monitoring | fears and worries |
| 40 | MoodKit | 4.7 | iPhone | Paid | Commercial  (Profit Organizations) | Health &fitness | September 27, 2016 | Personalization  Self-monitoring  Tunneling  Reminder | Negative emotions, Depression, Anxiety/Stress, Anger |
| 41 | iCBT | 4 | iPhone | Paid | Commercial  (Profit Organizations) | Medical | September 24, 2016 | Tunneling  Self-monitoring  Personalization | stress and anxiety |
| 42 | Moodnotes | 4.7 | iPhone | Paid | Commercial  (Profit Organizations) | Health &fitness | July 26, 2017 | Self-monitoring | distress and well-being |
| 43 | WorryWatch | No Rating | iPhone | Paid | Unknown | Health &fitness | January 22, 2018 | Self-monitoring  Reminder  Personalization | Negative emotions, Anxiety/Stress., fear, panic attacks, and depression |
| 44 | Stress Relief & Management App | 4.4 | iPhone | Free * | Commercial  (Profit Organizations) | Medical | February 20, 2018 | Nothing | Anxiety/Stress |
| 45 | Guided Meditation by Zen Mixer | 4 | iPhone | Free * | Commercial  (Profit Organizations) | Medical | March 14, 2018 | Expertise  Personalization  Reminder  rewards  Self-monitoring | Stress |
| 46 | HappiJar | 4.3 | iPhone | Free * | Unknown | Lifestyle | February 12, 2016 | Focus on past success  Personalization  Reminder  Tunneling | Increase Happiness/Well-being |
| 47 | ReachOut Breathe | 4.1 | iPhone | Free | Commercial  (Profit Organizations) | Health &fitness | December 20, 2015 | Personalization  Reminder | stress and anxiety |
| 48 | Thought Diary Pro | No Rating | iPhone | Paid | Commercial  (Profit Organizations) | Medical | September 30, 2014 | Expertise  Self-monitoring | Negative emotions |
| 49 | Cove | 4.3 | iPhone | Free | Commercial  (Profit Organizations) | Health &fitness | October 17, 2017 | Personalization  Verifiability  Self-monitoring  tunneling | Depression |
| 50 | Personal Zen | No Rating | iPhone | Free | Commercial  (Profit Organizations) | Health &fitness | June 25, 2016 | Focus on positive things  Reward  Self-monitoring | Anxiety/Stress |
| 51 | RealifeChange | 4.1 | iPhone | Free * | Commercial  (Profit Organizations) | Health &fitness | December 23, 2017 | Normative influence  Self-monitoring | moods disorder, stress, |
| 52 | Moods: Tracking For Better Mental Health | 3.7 | iPhone | Free | Commercial  (Profit Organizations) | Health &fitness | February 13, 2017 | Self-monitoring  Reminder  Personalization | Increase Happiness/Well-being |
| 53 | Stigma: Mood Tracker & Journal | 4.5 | iPhone | Free * | Commercial  (Profit Organizations) | Lifestyle | January 22, 2018 | Reminder  Self-monitoring  Normative influence | Mood disorders; Stress & Anxiety |
| 54 | Seven Minute Stress Cure | 2.4 | iPhone | Free | Unknown | Health &fitness | September 20, 2016 | Nothing | anxiety and depression |
| 55 | TruReach - Anxiety, Stress & Depression | 3.7 | iPhone | Free * | Commercial  (Profit Organizations) | Health &fitness | July 24, 2017 | Reward  Self-monitoring  Personalization | anxiety, stress, and feeling down. |
| 56 | Inspirational Happiness Tips! | 4.6 | iPhone | Free * | Commercial  (Profit Organizations) | Books | February 23, 2018 | Nothing | Increase Happiness/Well-being |
| 57 | eMoods Bipolar Mood Tracker | 4.8 | Android | Free * | Commercial  (Profit Organizations) | Lifestyle | May 8, 2018 | Self-monitoring  Personalization  Reminder | Depression, Anxiety, and mood disorders |
| 58 | Stop Panic & Anxiety Self-Help | 4.4 | Android | Free | Commercial  (Profit Organizations) | Medical | August 19, 2016 | Personlization | panic attack |
| 59 | Worry Box---Anxiety Self-Help | 2.7 | Android | Free | Commercial  (Profit Organizations) | Health &fitness | February 1, 2016 | Tunneling | Worry and Anxiety |
| 60 | Daily Feats | 3.9 | Android | Free | University | education | 5, Oct, 2017 | Personalization  Praise  Self-monitoring | general mental health |
| 61 | My Mantra | 4.2 | Android | Free | University | education | 3/23/2018 | Rehearsal  Personalization | depression and anxiety. |
| 62 | Mood Triggers: Anxiety Depression Insomnia Tracker | 3.9 | Android | Free | Unknown | Health &fitness | March 15, 2018 | Self-monitoring | anxiety or depression, stress |
| 63 | Worry Knot | 3.4 | Android | Free | University | Health &fitness | October 17, 2019 | Praise  Self-monitoring  Personalization | worry |
| 64 | Simple Habit-Meditation | 4.8 | Both | Free * | Commercial  (Profit Organizations) | Health &fitness | August 29, 2018 | Expertise  Reminder  Personalization  Social facilitation | stress and sleep better |
| 65 | Stop, Breathe & Think | 4.8 | Both | Free * | Commercial  (Profit Organizations) | Health &fitness | May 23, 2018 | Self-monitoring  Reward  Reminder  Personalization | stress |
| 66 | 10% Happier: Meditation | 4.8 | Both | Free * | Commercial  (Profit Organizations) | Health &fitness | July 18, 2018 | Nothing | improve well-being |
| 67 | Take a Break! - Meditations for Stress Relief | 4.8 | Both | Free * | Commercial  (Profit Organizations) | Health &fitness | June 8, 2017 | Nothing | stress |
| 68 | Zenfie Mindfulness Meditation | 4.6 | Both | Free * | Commercial  (Profit Organizations) | Health &fitness | July 12, 2016 | Self-monitoring  Real-world feel  Reminder | Stress |
| 69 | The Mindfulness App | 4.5 | Both | Free * | Commercial  (Profit Organizations) | Health &fitness | July 2, 2018 | Self-monitoring  Expertise  Personalization  Praise  Reduction  Reminder | Increase Happiness/Well-being |
| 70 | ThinkUp: Positive Affirmations | 4.7 | Both | Free * | Commercial  (Profit Organizations) | Health &fitness | April 30, 2018 | self-talk  Personalization  Reminder  Expertise | Depression, Anxiety |
| 71 | Antistress - relaxation toys | 4.7 | Both | Free * | Commercial  (Profit Organizations) | Games | July 3, 2018 | distraction | Stress |
| 72 | Aware - Meditation & Mindfulness | 4.8 | Both | Free * | Commercial  (Profit Organizations) | Health &fitness | June 17, 2018 | Expertise  Reminder  Self-monitoring | calm and sleep |
| 73 | Mental Health Intervention | 5 | Both | Free * | Unknown | Medical | December 6, 2016 | Self-monitoring  Real-world feel  Tunneling  Reminder | anxiety, depression |
| 74 | Project Toe | 2.3 | Both | Free | Commercial  (Profit Organizations) | Health &fitness | June 21, 2016 | Normative influence | depression, anxiety, PTSD, |
| 75 | Oprah & Deepak's 21-Day Meditation Experience | 3.5 | Both | Free * | Commercial  (Profit Organizations) | Health &fitness | July 28, 2018 | Reminder | reduce anxiety and stress  sleep better |
| 76 | DARE - Break Free From Anxiety | 4.5 | Both | Free * | Commercial  (Profit Organizations) | Health &fitness | April 12, 2018 | Nothing | negative emotions, Depression , Anxiety/Stress |
| 77 | Rootd - Panic Attack & Anxiety Relief | 4.3 | Both | Free * | Commercial  (Profit Organizations) | Health &fitness | February 2, 2018 | Self-monitoring  Praise  Rewards | panic attacks and anxiety |
| 78 | FREE Anxiety & Panic Relief | 3.6 | Android | Free | Commercial  (Profit Organizations) | Health &fitness | April 9, 2014 | Nothing | Panic, stress and anxiety |
| 79 | Feeling Good: positive psychology | 3.9 | Android | Free * | Commercial  (Profit Organizations) | Health &fitness | June 26, 2018 | Reminder  Self-monitoring | stress, depression, and anxiety |
| 80 | Relaxing Anti-Stress Sound | 4 | Android | Free | Unknown | Health &fitness | January 10, 2017 | Nothing | Anxiety, anger, depression |
| 81 | Moodfit - Stress & Anxiety | 4.4 | Android | Free | Commercial  (Profit Organizations) | Health &fitness | March 30, 2018 | Reminder  Self-monitoring  Goal setting | improve mood |
| 82 | Reachout: My Support Network | 4.4 | Android | Free | Commercial  (Profit Organizations) | Health &fitness | June 6, 2018 | Normative influence | Anxiety, Depression, , PTSD, Bipolar Disorder, |
| 83 | Wellness Hub | 4.5 | Android | Free * | Commercial  (Profit Organizations) | Health &fitness | February 15, 2018 | social role | Depression, Anxiety/Stress and Happiness/Well-being |
| 84 | Life Reboot - Fight Depression | 3.8 | Android | Free | Commercial  (Profit Organizations) | Health &fitness | October 28, 2015 | Normative influence  Reminder  distraction | general mental health |
| 85 | overcome the depression | No Rating | Android | free | Unknown | Health &fitness | January 5, 2015 | Reminder | depression |
| 86 | Cognitive Diary CBT Self-Help | 4.3 | Android | Free | Commercial  (Profit Organizations) | Medical | November 7, 2016 | Personalization  Reminder | Depression, Anxiety/Stress and Increase Happiness/Well-being |
| 87 | Beat Depression | 3.9 | Android | Free | Unknown | Adventure | July, 15 2019 | Expertise  focus on important things | Depression |
| 88 | Depressed | 4.3 | Android | Free | Unknown | entertainment | March 26, 2018 | Nothing | depression |
| 89 | MoodSpace | 4.6 | Android | Free * | Commercial  (Profit Organizations) | Medical | July 17, 2018 | Nothing | depression |
| 90 | Daylio - Diary, Journal, Mood Tracker | 4.8 | Android | Free * | Commercial  (Profit Organizations) | Lifestyle | May 15, 2018 | Self-monitoring  Reminder  Personalization | Increase Happiness/Well-being |
| 91 | Wysa - your happiness buddy | 4.7 | Android | Free * | Commercial  (Profit Organizations) | Health &fitness | June 8, 2018 | social role  Self-monitoring | stress, anxiety, sleep |
| 92 | Vent - Express yourself freely | 4.6 | Android | Free * | Commercial  (Profit Organizations) | social networking | July 24, 2018 | Self-monitoring  Social facilitation  Normative influence | general mental health |
| 93 | Depression CBT Self-Help Guide | 4.2 | Android | Free | Commercial  (Profit Organizations) | Medical | November 3, 2016 | Self-monitoring  rewards  Personalization | Stress |
| 94 | Perspective - Daily Journal | 4.7 | iPhone | Free | Commercial  (Profit Organizations) | Lifestyle | August 17, 2017 | Self-monitoring  Reminder  Tunneling  Personalization | mental health, and well-being |
| 95 | Mindfulness Daily | 4.6 | iPhone | Free * | Commercial  (Profit Organizations) | Health &fitness | January 23, 2018 | Personalization  Reminder  Self-monitoring  Expertise  Real-world feel | stress/anxiety, better sleep |
| 96 | 3 Minute Mindfulness | 4.3 | iPhone | Free * | Commercial  (Profit Organizations) | Health &fitness | June 9, 2018 | Reminder  Self-monitoring  Personalization | stress, anxiety, overwhelm, sleep |
| 97 | Breathe+ Simple Breath Trainer | 4.8 | iPhone | Free * | Commercial  (Profit Organizations) | Health &fitness | February 20, 2018 | Personalization  Tunneling  Self-monitoring  Reminder | general mental health, stress |
| 98 | Free Relaxing Nature Scenes to Reduce Stress & Anxiety | 4.2 | iPhone | Free | Commercial  (Profit Organizations) | Lifestyle | August 24, 2016 | Personalization | Stress, Sleep and feel better, |
| 99 | Mood Mint' Boost Your Mood | 4.6 | iPhone | Paid | Unknown | Health &fitness | July 1, 2015 | Personalization  Reward  distraction | stress, anxiety and depression. |
| 100 | Relax Now: Hypnosis Meditatio | 4.6 | iPhone | Free | Unknown | Medical | April 30, 2018 | Personalization | stress |
| 101 | Anxiety Relief Hypnosis PRO | 4 | iPhone | Paid | Commercial  (Profit Organizations) | Medical | Jun 17 2018 | Nothing | General mental health |
| 102 | Moodtrack Social Diary | 4.5 | iPhone | Free * | Unknown | Lifestyle | February 23, 2018 | Self-monitoring  Social facilitation  Reminder  Normative influence | Happiness/Well-being |
| 103 | Oak - Meditation & Breathing | 4.7 | iPhone | Free | Commercial  (Profit Organizations) | Health &fitness | December 12, 2017 | Personalization  Self-monitoring  Reminder , reward | General mental health |
|  | Free * = Free with in-app purchases | | | | | | | | |
